# Association of Dietary Patterns, Circulating Lipid Profile, and Risk of Obesity

**DOI:** 10.1053/j.gastro.2023.03.233

**Published:** 2023-04-13

**Authors:** Lang Pan, Kexiang Shi, Jun Lv, Yuanjie Pang, Yu Guo, Pei Pei, Huaidong Du, Iona Millwood, Ling Yang, Yiping Chen, Ruqin Gao, Xiaoming Yang, Daniel Avery, Junshi Chen, Canqing Yu, Zhengming Chen, Liming Li, on behalf of the China Kadoorie Biobank Collaborative Group

**Affiliations:** 1 Department of Epidemiology & Biostatistics, School of Public Health, Peking University, Beijing 100191, China; 2 Peking University Center for Public Health and Epidemic Preparedness & Response, Beijing 100191, China; 3 Key Laboratory of Molecular Cardiovascular Sciences (Peking University), Ministry of Education, Beijing, China; 4 Fuwai Hospital Chinese Academy of Medical Sciences, National Center for Cardiovascular Diseases, Beijing, China; 5 Chinese Academy of Medical Sciences, Beijing, China; 6 Medical Research Council Population Health Research Unit at the University of Oxford, Oxford, United Kingdom; 7 Clinical Trial Service Unit & Epidemiological Studies Unit (CTSU), Nuffield Department of Population Health, University of Oxford, United Kingdom; 8 Qingdao Municipal Center for Disease Control and Prevention, Qingdao, China; 9 China National Center for Food Safety Risk Assessment, Beijing, China

**Keywords:** dietary pattern, lipid metabolism, overweight, general obesity, central obesity

## Abstract

**Objective:**

To simultaneously explore the associations of major dietary patterns with lipid profiles and of these profiles with general and central obesity risks and to evaluate the extent to which these metabolites mediate such associations.

**Methods:**

Habitual foods consumption of 4,778 participants with an average age of 47.0 from the China Kadoorie Biobank was collected using a 12-item food frequency questionnaire. Plasma samples were analyzed via targeted NMR spectroscopy to quantify 129 lipid-related metabolites. Anthropometric information was measured by trained staff.

**Results:**

Two dietary patterns were derived by factor analysis. The newly affluent southern pattern was characterized by high intakes of rice, meat, poultry, and fish, whereas the balanced pattern was characterized by consuming meat, poultry, fish, fresh fruit, fresh vegetables, dairy, eggs, and soybean. The newly affluent southern pattern was positively associated with 45 metabolites, which were positively associated with risks of obesity at the same time. The global lipid profile potentially explained 30.9%, 34.7%, and 53.1% of the effects of this dietary pattern on general obesity, WC-defined central obesity, and WHR-defined central obesity, respectively.

**Conclusions:**

The newly affluent southern pattern points to an altered lipid profile, which showed higher general and central obesity risks. Our findings partly suggest the biological mechanism for the obesogenic effects of this dietary pattern.

## Introduction

Obesity is a chronic disease and an independent risk factor of many major diseases.(1) Over the past 30 years, the prevalence of overweight, general, and central obesity soared in both men and women, globally and in China.(2, 3, 4)

Unhealthy dietary habit is a well-established risk factor for obesity.(5) Dietary patterns (DPs), rather than single nutrients, are gaining more attention since each dietary habit is a complex system consisting of multiple foods. DPs can provide a more holistic view of food combinations, synergies, and antagonisms and may establish dietary recommendations that are easier to adopt for the public.(1, 6, 7) Substantial evidence from observational and interventional studies has suggested that several DPs, such as the Mediterranean diet and dietary approaches to stop hypertension (DASH), are significantly associated with obesity.(8, 9). In addition, although investigator-derived DPs are the most widely used, they are limited to existing health experience and may not represent the most appropriate scientific evidence compared with data-derived DPs, also known as the posterior approach.(1, 6)

The pathophysiology of obesity is complex and ambiguous.(10) Lipid metabolism, closely related to specific DPs and their major food groups, is also considered one of the crucial obesogenic pathways.(11, 12, 13) The relationship between DP, lipid metabolism, and obesity has always been a hot topic, and many research conclusions have erupted. Regrettably, few studies have explored these associations in the same population, which may provide more homogeneous evidence. Furthermore, lipid-related metabolites, especially lipoproteins, can be divided into subclasses of different densities and sizes, such as large high-density lipoprotein (large HDL) and small low-density lipoprotein (small LDL), and so on.(13, 14) Total cholesterol (TC), triglyceride (TG), apolipoprotein, and other subfractions within different lipoproteins are almost impossible for traditional biochemical approaches to detect.(15)

The present study aimed to simultaneously explore the associations of data-derived DPs with plasma lipid profiles and of these profiles with risks of overweight, general, and central obesity in the China Kadoorie Biobank (CKB).

## Methods

### Participants and study design

A total of 512,725 adults from 10 areas in China were enrolled in the CKB study between 2004 and 2008.(16) These eligible participants were all permanent residents of the region aged 30-79 years, without severe physical disabilities and able to communicate normally, and their disease and death registration reports were managed by the local health authorities. Baseline information, including a laptop-based questionnaire and physical measurement, was collected after signing a written informed consent form. All participants provided a 10mL non-fasting blood sample for long-term storage, with time since the last meal recorded. In addition, about 5% of the participants were randomly selected to join the resurvey every 5 years after completing the baseline survey.

The present study included 4,778 participants with metabolomics data from a previous nested case-control study based on the CKB.(17) Briefly, cases were incident cases of myocardial infarction (MI), ischaemic stroke (IS), or intracerebral haemorrhage (ICH) with a censoring date of 1 January 2015. Common controls were frequency matched, where possible, to the combined cases by age, sex, and area, and were alive and free of stroke of any type, MI, or CHD by the censoring date. All participants had no history of self-reported prior doctor-diagnosed coronary heart disease (CHD), stroke, transient ischemic attack, or cancer. They were also not using statin therapy at baseline. The Ethical Review Committee of the Chinese Center for Disease Control and Prevention (China, 005/2004) and the Oxford Tropical Research Ethics Committee of the University of Oxford (UK, 025-04) approved the study.

### Assessment of food consumption

With the brief food frequency questionnaire (FFQ) at baseline, participants were asked about their habitual frequency of 12 major food groups during the past 12 months, including rice, wheat, other staples, meat, poultry, fish, eggs, dairy, fresh fruit, fresh vegetable, preserved vegetable, and soybean. Possible answers were “never/rarely, monthly, 1-3 days per week, 4-6 days per week, and daily”. The frequency was then converted into weekly days of food consumption, with each option corresponding to 0, 0.5, 2, 5, and 7 days per week, respectively. In the 2^nd^ resurvey (2013-2014), participants were additionally asked about their daily amount when consuming the above foods. A validated picture booklet, which provided brochures of common units (such as a small bowl of rice or a chicken leg) of each food group and its corresponding weight, was used to assess portion sizes.

A separate validation study of the FFQs was conducted among 432 CKB participants.(18) Two FFQs and twelve 24-h dietary recalls (24-HDR) were used to evaluate the reproducibility and validity, respectively. For reproducibility, the weighted Kappa statistics were 0.62-0.90 for baseline food frequency and 0.60-0.82 for consumption amount in the 2^nd^ resurvey. The weighted Kappa statistics were 0.60-0.88 and 0.60-0.86 for relative validity of food frequency at baseline and consumption amount in the 2^nd^ resurvey, respectively.

A total number of 23,974 participants from the 2^nd^ resurvey, who were free of cardiovascular diseases (CVDs) and cancer and did not use statins, were stratified by sex, age group (<60y or ≥60y), region (10 regions), and food consumption group (never/rarely, monthly, 1-3 d/w, 4-6 d/w, or daily). The average daily amount in each stratum was calculated as a proxy to impute the amount of food consumption at baseline.(19)

### Plasma NMR metabolomics

Baseline plasma samples were couriered to Oxford for long-term storage in liquid nitrogen tanks after centrifuging and aliquoting. The stored plasma samples were thawed and sub-aliquoted at the Wolfson laboratory, CTSU. Then 100uL aliquots were shipped on dry ice to the Brainshsake Laboratory at Oulu, Finland, for high-throughput targeted NMR spectroscopy to quantify 129 lipid-related metabolites, including lipoproteins, fatty acids, and ketone bodies.(15)

### Anthropometric measurements and definitions

At baseline, trained staff collected anthropometric measurements according to standard procedures.(20) Weight was measured without shoes using the TBF-300GS Body Composition Analyser (Tanita Inc., Tokyo, Japan), accurate to 0.1 kg. The weight of clothing was estimated depending on the season and deducted from measured body weight. Standing height was measured without shoes using a stadiometer to the nearest 0.1 cm. Waist circumference (WC) and hip circumference were measured with a soft tape, also accurate to 0.1 cm. BMI was calculated as weight (kg) divided by height square (m^2^). The waist-to-hip ratio (WHR) was calculated as WC divided by hip circumference.

According to the guidelines for prevention and control of overweight and obesity in Chinese adults, overweight and general obesity were defined as BMI 24.0-<28.0 and BMI≥28.0 kg/m^2^, respectively.(21, 22) Central obesity was defined using two criteria according to previously recommended cut-off points.(22, 23) One was WC≥85 cm for men or WC≥80 cm for women. The other was WHR≥1.0 for men or WHR≥0.9 for women.

### Assessment of covariates

Information on sociodemographic characteristics (age, sex, region, education, household income, occupation, and marital status), lifestyle factors (tea-drinking habit, smoking status, alcohol intake, and physical activity), health status (hypertension, diabetes, and self-rated health scores), and family history (diabetes and CVD) was obtained from the baseline questionnaire. Fasting time, which was the time since the last meal, was asked before plasma collection.

### Statistical analysis

Based on the imputed weekly amount of 12 food groups consumption, DPs were derived using factor analysis with a principal component method. The factors were then rotated by orthogonal transformation to maintain the independent factors and greater interpretability.(24) The number of factors (DPs) was determined according to eigenvalue (>1), scree plot, variance explained (>10%), and factor interpretability. The DPs were named according to the food groups showing high absolute loadings (>0.40). The factor scores for each DP of each participant were calculated by summing the consumption of each food group weighted by its factor loading.

Baseline characteristics were presented as means or percentages across quartile categories of DP scores, adjusted by age, sex, and study region if appropriate, using multiple linear regressions for continuous variables or logistic regressions for categorical variables. For each lipid-related metabolite, measurements rejected by quality control were imputed with the average available concentration, while measurements below the limit of detection were imputed with half of the lowest available concentration.

Linear regression was used to assess the associations of DP scores with lipid-related metabolites, adjusted for age (years, continuous), sex (female or male), region (10 areas), education (junior high school and above, or not), household income (annual household income above 35,000 Yuan, or not), occupation (manual, non-manual, or others), marital status (married or not), tea-drinking habit (weekly tea drinker or not), smoking status (never/occasional smoker, former smoker, ≤10, ≤20, or more per day), alcohol intake (never/occasional drinker, former drinker, ≤25g for men or ≤15g for women, ≤50g for men or ≤30g for women, or more per day), physical activity (MET-h/d, continuous), self-rated health (poor or not), fasting time (hours, continuous), personal history of hypertension or diabetes, family history of diabetes or CVD, and groups of participants in the original case-control study. In order to reduce the possible confounding effect among the derived DPs, when considering one DP, the model included the other DP(s) as covariables, although the factors are theoretically orthogonal. Adjusted standard deviation (SD) differences and 95% confidence intervals (CI) associated with SD higher DP scores for each metabolite were estimated.

Logistic regression was performed to yield the odds ratios (ORs) for overweight, general obesity, WC-defined, and WHR-defined central obesity per SD higher lipid-related metabolites, with the same variables adjusted for as in the linear regression between DPs and metabolites. Underweight participants (BMI<18.5 kg/m^2^) were excluded from the metabolites-general obesity analysis. ORs were then plotted against SD differences in corresponding metabolites per SD higher DP scores.

In addition, we conducted mediation analyses to estimate the extent of the lipid profiles that potentially explained the association between DPs and obesity by adding 7 principal components (PCs, as mediators) that explained ≥95% of the variation across the 129 traits to the basic model and calculated the percent change in the ORs for obesity per SD higher DP scores. The proportion of obesity risk explained by the 7 PCs was calculated as: ((OR_basic model_ - OR_adjusted model_) / (OR_basic model_ - 1))*100%.(25) Bootstrapping analysis was deployed to obtain sub-samples and test whether 95%CIs of the mediating effect contain 0.

To examine the confounding effects of other suspicious factors and to assess the robustness of the results, we conducted several sensitivity analyses, including: (1) additionally adjusting daily energy intake calculated by daily total food intake, (2) adjusting significant weight changes over the past year (about the same as before, gained >2.5 kg, or lost >2.5 kg) and weight loss attempts (yes or no), and (3) excluding those participants whose weight changed significantly over the past year or those who tried to reduce weight in the past year (n=1,234).

All *p-*values were two-sided, and statistical significance was defined as *p*<0.05. To account for a large number of highly correlated metabolites, those that met the Bonferroni-corrected threshold [*p*<0.05/129/(*f*+4)] were considered significant, where *f* represented the number of DPs we derived. Statistical analyses were performed using Stata 15.0.

## Results

We derived two DPs. The first, which represented high intakes of rice, meat, poultry, and fish, but low intakes of wheat and other staples like corn and millet, was named as the newly affluent southern pattern. The second DP was named the balanced pattern, characterized by consuming meat, poultry, fish, fresh fruit, fresh vegetables, dairy, eggs, and soybean. These first two DPs explained 42.9% of the variability (Table 1 and Figure S1).

Among the 4,778 participants, the mean (SD) age was 47.0 (8.2) years, 50.1% were women, and 29.0% resided in urban areas. Compared to participants with a lower score of the newly affluent southern pattern (Q1), those with higher scores were younger, more likely to be male, from urban and southern areas, with higher annual household income but lower physical activities and education levels. For the balanced pattern, participants with higher scores were younger males as well, but from northern areas, with higher education levels, lower SBP, and lower diagnosed hypertension (Table 2).

**Figure 1:**
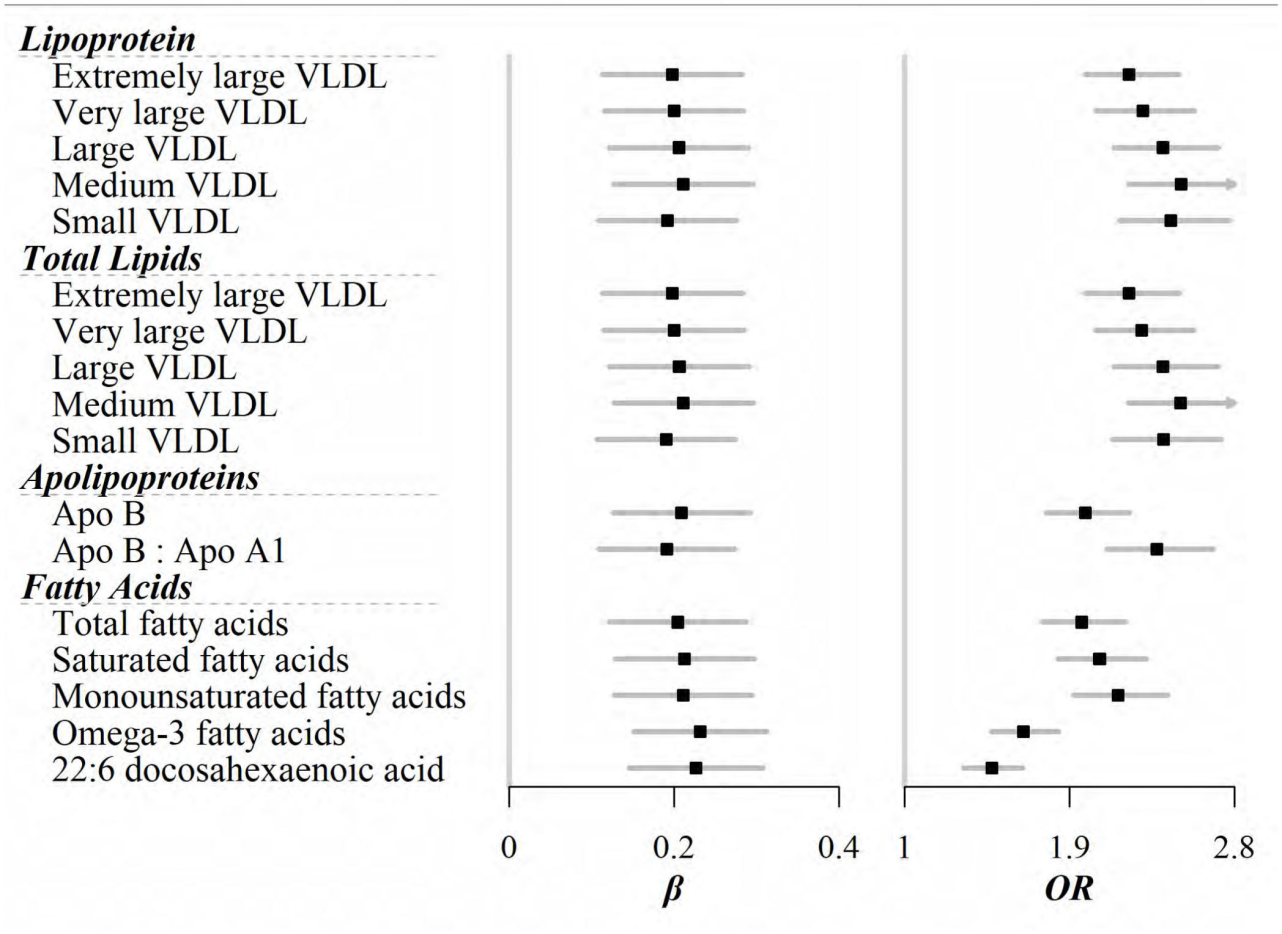
Lipid-related metabolites that were significantly associated with the newly affluent southern pattern scores [*β* coefficient (95%CI)]. Further, these metabolites were all associated with risk of general obesity [OR (95%CI)]. Models were adjusted for age, sex, region, education, household income, occupation, marital status, tea-drinking habit, smoking status, alcohol intake, physical activity, self-rated health, fasting time, and groups of participants in the original case-control study. All analyses relating to one DP were adjusted for others. Black squares represented coefficients or ORs, while gray horizontal lines represented 95%CIs.

**Figure 2:**
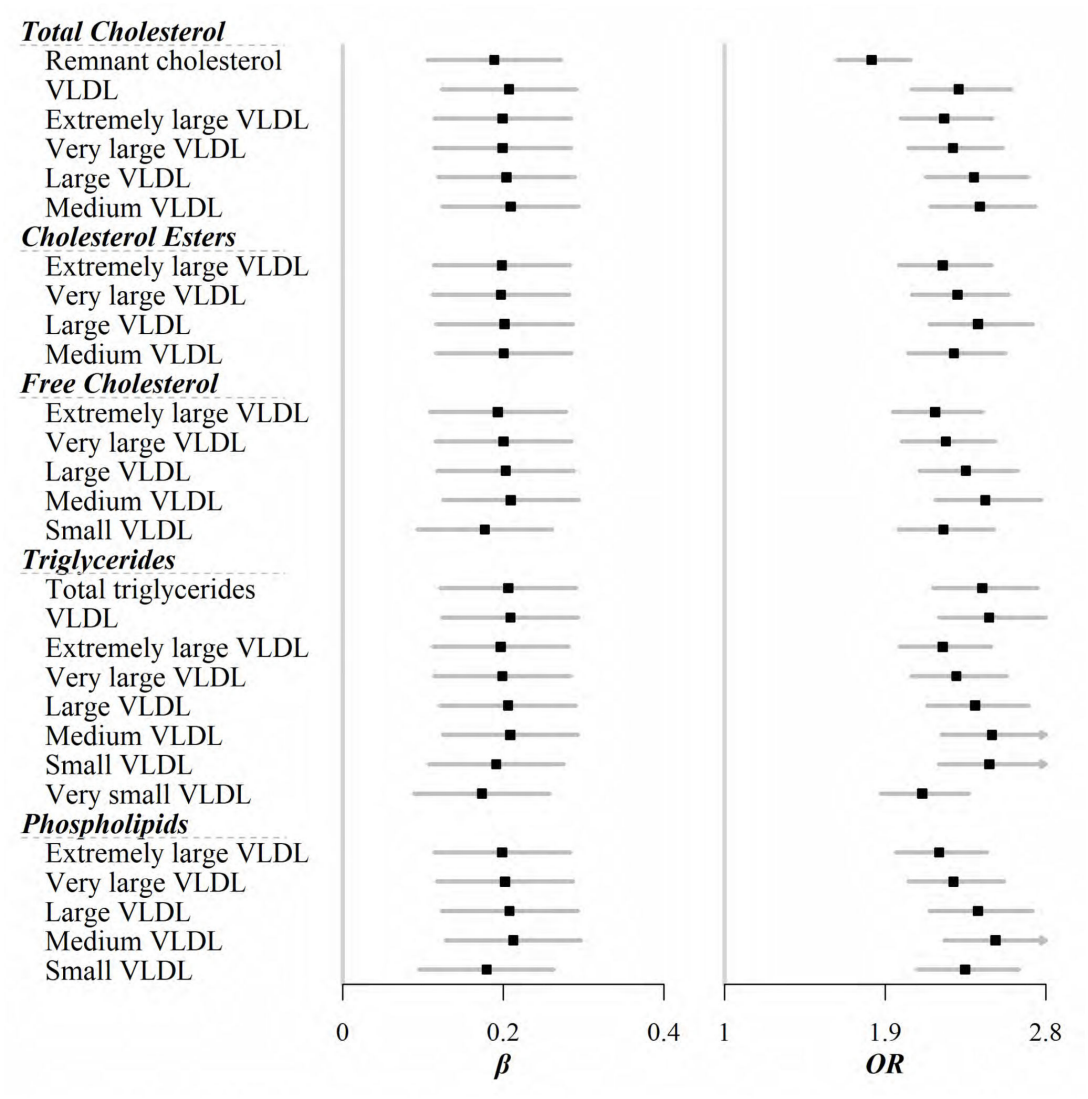
Lipoprotein subfractions that were significantly associated with the newly affluent southern pattern scores (*β*). Further, these metabolites were all associated with risk of general obesity (OR). Models were adjusted for age, sex, region, education, household income, occupation, marital status, tea-drinking habit, smoking status, alcohol intake, physical activity, self-rated health, fasting time, and groups of participants in the original case-control study. All analyses relating to one DP were adjusted for others. Black squares represent coefficients or ORs, while gray horizontal lines represent 95%CIs.

After Bonferroni correction, 45 out of 129 lipid-related metabolites still showed positive associations with the newly affluent southern pattern (Figure 1, Figure 2, and Table S1). For lipoproteins, five different sizes of very-low-density lipoprotein (VLDL) were implicated in this DP. For each SD increase of the newly affluent southern pattern score, VLDL with most of particle sizes increased by about 0.2 SD except very small VLDL [ORs (95%CIs) were 0.20 (0.11, 0.28), 0.20 (0.12, 0.28), 0.21 (0.12, 0.29), 0.21 (0.13, 0.30), and 0.19 (0.11, 0.28) for extremely large, very large, large, medium, and small VLDL, respectively]. Total lipids within these VLDL particles showed similar associations (Figure 1). When focused on specific lipoprotein subfractions, not surprisingly, lipids including TC, cholesterol esters, free cholesterol, TG, and phospholipids within most of VLDL, except for a few smaller sizes, were correlated with this DP (Figure 2). Apolipoprotein B, along with its ratio to A1, as well as fatty acids, no matter saturated and unsaturated, all had higher levels in participants who complied with the newly affluent southern pattern (Figure 1). However, no metabolite correlated with the balanced pattern on the Bonferroni-corrected level (Table S2).

The prevalence of overweight, general obesity, WC-, and WHR-defined central obesity was 34.70%, 13.06%, 44.24%, and 19.49%, respectively. Among 129 metabolites, 108, 90, 107, and 99 were related to the corresponding risks (Table S3 and S4). More importantly, all 45 metabolites, which were associated with the newly affluent southern pattern, showed correlations with overweight and obesity at the same time, even after the Bonferroni adjustment. For example, the strongest association was seen for phospholipids within medium VLDL, with each SD increase associated with 105%, 152%, 145%, and 85% increase in the risk of overweight, general obesity, WC-, and WHR-defined central obesity, respectively.

**Figure 3:**
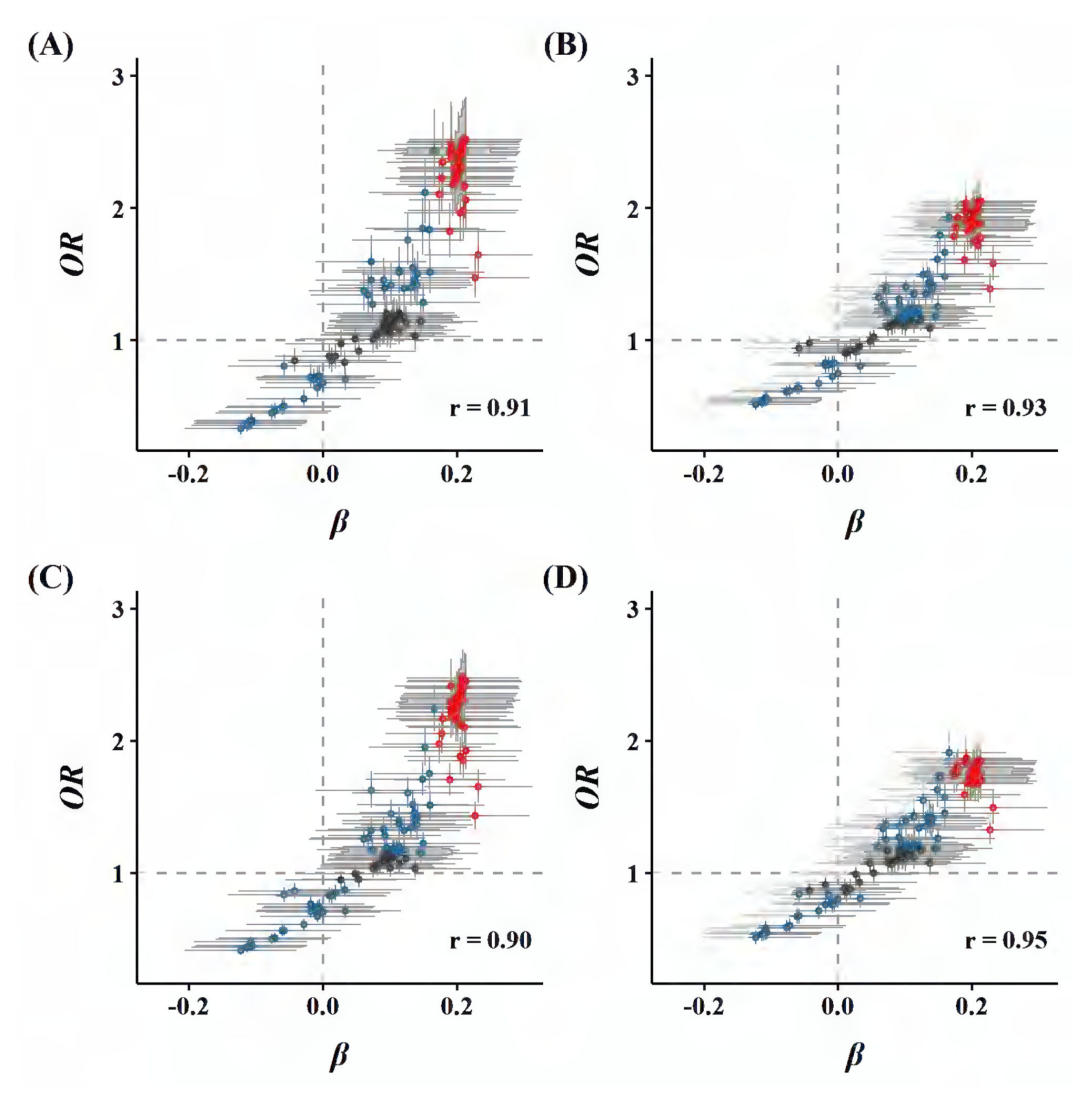
Global comparisons of *β* coefficients for metabolites and newly affluent southern pattern vs. ORs for (A) general obesity, (B) overweight, (C) WC-defined central obesity, and (D) WHR-defined central obesity with these metabolites. Models were adjusted for age, sex, region, education, household income, occupation, marital status, tea-drinking habit, smoking status, alcohol intake, physical activity, self-rated health, fasting time, and groups of participants in the original case-control study. All analyses relating to one DP were adjusted for others. Blue dots represented metabolites associated with the risk of obesity but not with the newly affluent southern pattern, while red dots represented metabolites associated with both the newly affluent southern pattern and the risk of obesity, with overlapping dots darker in color. The gray horizontal line and vertical line represented 95%CIs of coefficients and ORs, respectively. Pearson correlations of coefficients and ORs were annotated in the lower right corner.

The newly affluent southern pattern showed positive associations with risks of overweight, general, and central obesity [ORs per SD higher DP score were 1.42 (1.15, 1.76), 1.82 (1.34, 2.48), 1.47 (1.21, 1.79), and 1.36 (1.04, 1.78), respectively]. There was consistency in the direction between the associations of the newly affluent southern pattern with lipid profiles and of these profiles with risk of obesity. In other words, when this DP was associated with altered levels of specific lipid-related metabolite, corresponding altered levels were associated with higher risks of overweight, general, and central obesity (Pearson correlation coefficients were 0.91, 0.93, 0.90, and 0.95, respectively; Figure 3).

In sensitivity analyses, the association patterns between the newly affluent southern pattern, lipid profiles, and obesity risks kept unchanged when (1) additionally adjusting daily energy intake calculated by daily total food intake, (2) adjusting significant weight changes over the past year (about the same as before, gained >2.5 kg, or lost >2.5 kg) or weight loss attempts (yes or no), and (3) excluding those participants whose weight changed significantly over the past year or those who tried to reduce weight in the past year (Figure S2-S4, respectively).

Further mediation analyses showed that the first 7 principal components, which explained ≥95% of the variation across 129 lipid-related metabolites, potentially explained 30.9%, 34.7%, and 53.1% of the association of the newly affluent southern pattern with risks of general, WC-defined, and WHR-defined central obesity, respectively (*p*_*mediation*_<0.05).

## Discussion

In summary, our study identified a newly affluent southern pattern in a Chinese population, which was associated with 45 metabolites including TC, cholesterol esters, free cholesterol, TG, and phospholipids. Others were Apolipoprotein B and a series of fatty acids. Moreover, these 45 metabolites all showed positive correlations with risks of overweight, general, and central obesity at the same time. The global difference in 129 metabolites related to higher compliance of this DP conferred higher risks of general and central obesity, with 30.9% and 34.7% explained by these metabolites.

Based on 12 major food groups consumed in the Chinese population, these DPs we constructed could reflect, to some extent, the transitions in nutrition and dietary habits in China during the recent decades caused by economic, social, and cultural changes. They were consistent with previously reported results from large-scale studies from the Chinese population.(26, 27) Additionally, the newly affluent southern pattern was in line with the temporal trend of increasing fat and decreasing coarse grains captured by the China Health and Nutrition Survey (CHNS), which covered most provinces and autonomous regions in China.(28, 29)

The most crucial DP we derived was the newly affluent southern pattern, explaining more than a fifth of the variation in 12 food groups. This DP may be partly to blame for obese status. Participants following this pattern showed higher levels of VLDL and its lipid subfractions. One explanation was the role of a high intake of refined rice but a low intake of whole grains. A previous review summarized increasing TG and TG-riched lipoproteins (VLDL and LDL) after refined carbohydrate intake.(30) On the other hand, two meta-analyses based on 25 and 66 RCTs showed that whole grain intake reduced TC, TG, and LDL levels.(31, 32) In addition, the harmful changes to TC and TG within VLDL by red meat intake have long been reported, in line with the heavy loading of red meat on the newly affluent southern pattern.(33) A combination of high intakes of refined carbohydrates and saturated fatty acids, similar to the newly affluent southern pattern, found increased VLDL cholesterol in animal models, which was also directionally consistent with our findings.(34) The possible mechanism was that this dietary habit induced changes in VLDL receptors, which promoted the transfer of dietary lipids from skeletal muscle to adipose tissue and inhibited the clearance of TG-rich lipoproteins.

Recently a study based on the larger CKB population reported that a DP similar to the newly affluent southern pattern was associated with cardiometabolic risk.(35) In the present study, the NMR spectroscopy allowed us to distinguish different particle sizes of lipoproteins. Smaller and more dense VLDL was more likely to deposit in the arteries and hence was always considered more diabetic and atherogenic.(36) This was similar to the association between VLDL and obesity risk in the present study, with smaller VLDL and its subfractions appearing to have more significant effects on obesity (Figure 1 and 2).

People who followed the newly affluent southern pattern also consumed more nutritionally defined white meat, such as poultry and fish. A study of more than 26,000 US women using the 131-item FFQ found that habitual fish consumption reduced VLDL and TG in VLDL levels and increased HDL cholesterol levels.(37) Similar results had been reported in intervention studies.(38) Inconsistencies regarding the effects of white meat on plasma lipids might be attributable, in part, to consumption habits for different species of fish.(39) Moreover, participants in our study consumed 2-3 times more red meat than white meat, which might mask the latter effect. The complex effects of food combinations might compromise the effect of each food group, such as refined rice, red meat, and fish, which did reflect the advantage of analyzing dietary patterns rather than separate food groups.

These lipid metabolites accounted for about one-third of the harmful effects of this pattern on both general and central obesity, suggesting that there might be several other obesogenic pathways. For instance, a cross-sectional study based on 145 Asian Indians identified a DP called the Western/nonvegetarian pattern.(40) It featured high intakes of red meat, poultry, fish, and pizza, much similar to our newly affluent southern pattern, and was positively correlated with branched-chain amino acids, aromatic amino acids, and short-chain acylcarnitine. Other pathways might include inflammation and phenolic metabolism.(41)

Given the same BMI or body fat mass, the Chinese population was found to have more body fat percentage and visceral adipose tissue than the European population.(42) In this context, in addition to choosing the established WC cutoff, we also defined central obesity according to WHR as a comparison. The correlation of WHR-defined central obesity with a specific metabolite was substantially weaker than WC-defined central adiposity (Figure 3). That was possibly due to the controversial WHR cutoff among Asians, especially Chinese, and our stricter cutoff selection.

More and more countries have recommended a healthy dietary pattern rather than specific foods in their dietary guidelines in recent years. The latest Dietary Guidelines for Americans 2020-2025 placed “following a healthy dietary pattern across the lifespan” relatively high priority. The Chinese Nutrition Society recommended improving and maintaining overall health through adopting healthier dietary patterns and visualized a Food Guide Pagoda for Chinese residents.(43) As a relatively unhealthy dietary pattern against established recommendations, the newly affluent southern pattern derived in the present study extended evidence of its obesogenic effect through the lipid metabolism pathway.

Our study is one of the largest studies that considered various sizes and subfractions of lipid profiles into the association between habitual diet consumption and obesity risk based on the Chinese population. However, a few limitations are worth noting when interpreting our findings. First, the FFQ was limited to the 12 major food groups. We did identify DPs that could capture the dietary habits of Chinese residents to a certain extent and were similar to previously reported DPs using broader food items. However, other food groups that may be related to general or central obesity were not considered, such as oil, sugar-sweetened beverages, and seasonings like sugar and salt. The lack of detailed food processing information also makes dietary habits more obscure. Besides, since the baseline FFQ only included consumption frequency information, we imputed the amount of each stratum using the approach used in previous studies, which would introduce information bias. Second, the number of DPs was determined subjectively. We derived two DPs that explained less than half of the variation together. The remaining half of the variation may be in minor patterns that we have not derived, possibly pointing to specific lipid profiles and obese risk. Third, albeit many potential sociodemographic and behavioral confounding factors were adjusted for in our multivariable models, residual confounding due to uncollected or suboptimally collected factors may still exist. Fourth, although participants were free of CVD or cancer, did not use statin therapy, and answered about their dietary habits over the past year rather than the last few days, the cross-sectional nature limits causal inference. Therefore, longitudinal studies will be complementary to the present study.

## Conclusion

To sum up, the newly affluent southern dietary pattern was associated with higher concentrations of apolipoprotein B, fatty acids, VLDL of different particle sizes, and their subfractions. In general, these lipid profiles were associated with higher risks of overweight, general, and central obesity. Our study provides cross-sectional evidence of the lipid metabolism pathway between this pattern and the obese risk. Longitudinal and intervention studies using higher abundance quantification technology are needed to clarify and extend the causal roles of metabolites in such associations.

## Supplementary Material

Supplementary File

## Figures and Tables

**Table 1:** Factor loading matrix of 12 food groups on major DPs by principal component analysis with varimax rotation. Loadings in bold indicated absolute values >0.40. DP3 was excluded from further analysis because only two food groups, preserved vegetables and other staples, loaded heavily on this pattern.

Food group	Newly affluent southern pattern	Balanced pattern	Dietary pattern 3
Rice	**0.91**	-0.05	0.08
Wheat	**-0.86**	0.10	0.19
Other staples	**-0.56**	-0.08	**-0.63**
Meat	**0.54**	**0.50**	0.15
Poultry	**0.47**	**0.53**	-0.07
Fish	**0.47**	**0.48**	-0.05
Eggs	-0.17	**0.50**	-0.09
Fresh vegetable	-0.17	**0.66**	0.02
Preserved vegetable	-0.14	0.02	**0.87**
Dairy	-0.08	**0.60**	-0.01
Soybean	-0.06	**0.54**	0.22
Fresh fruit	-0.02	**0.59**	0.23
Eigenvalue	2.70	2.45	1.34
Variance explained (%)	22.51	20.40	11.17

**Table 2:** Characteristics of participants by quartile of major dietary patterns. Abbreviations: CNY, China Yuan; BMI, body mass index; WC, waist circumference; WHR, waist-to-hip ratio; SBP, systolic blood pressure; MET, metabolic equivalent.

Characteristics	Newly affluent southern pattern			Balanced pattern		
	Q1	Q4	*P*_trend_	Q1	Q4	*P*_trend_
N	1,196	1,194		1,195	1,194	
Age, years	48.35	44.82	<0.01	48.65	45.71	<0.01
Female, %	40.43	24.89	<0.01	68.52	34.63	<0.01
Urban area, %	2.85	30.70	<0.01	1.80	75.48	<0.01
Southern area, %	0.00	97.81	<0.01	56.05	28.80	<0.01
Middle school or above, %	56.93	51.46	0.02	48.45	66.81	<0.01
Household income ≥35000	3.44	17.22	<0.01	4.23	21.41	<0.01
CNY/year, %						
Manual worker, %	72.47	67.10	0.02	79.58	58.48	<0.01
BMI, kg/m^2^	23.40	24.20	0.05	23.85	24.21	0.03
WC, cm	80.10	81.50	0.30	80.84	81.90	0.02
WHR	0.88	0.89	0.06	0.89	0.89	0.70
SBP, mmHg	136.05	137.55	0.79	140.87	134.45	<0.01
Regular smoker, %	37.87	36.82	0.93	38.84	35.62	0.09
Weekly drinker, %	10.89	23.46	<0.01	14.74	21.11	<0.01
Physical activity, MET-h/day	25.15	21.43	<0.01	23.03	21.56	0.04
Fasting time, h	4.28	4.24	0.82	14.24	16.24	0.14
Diabetes, %	6.35	5.13	0.50	6.08	8.18	0.07
Hypertension, %	41.83	45.57	0.63	49.15	42.57	0.01
Family history of diabetes, %	7.17	10.96	0.04	7.05	9.62	0.06

## Data Availability

Details of how to access China Kadoorie Biobank data and details of the data release schedule are available from www.ckbiobank.org/site/Data+Access.
